# On a Method of Infant-Feeding by Home-Made Humanised Milk

**Published:** 1904-01-09

**Authors:** C. Beauchamp Hall


### ON A METHOD OF INFANT-FEEDING BY HOME-MADE HUMANISED MILK.

By C. BEAUCHAMP HALL, F.R.C.S.Eng.

THE question of infant-feeding has always been an important one, and more especially ought we to make careful study of it at the present time on account of the daily increase in the number of women who will not nurse their offspring. The future health of many an infant is sacrificed from this cause yearly.

In selecting a food for the hand-reared infant let us imitate nature as far as we can. Nature provides the mother with an ideal infant's food—ideal not only in composition, but also in possessing that inherent quality of all natural foods, the indescribable element of freshness. What this element is we know not, nor can we manufacture it and sell it to the general public in a bottle. But it is the essential part of an infant's food, the one element above all others that prevents the appearance of scurvy and rickets. If, then, we have to make an artificial food we must borrow this fresh element from another source, and cow's milk will conveniently form that source. Now from cow's milk we can make a food almost if not actually identical with mothers' milk, and the chief question that arises is how to do so. I believe the following method to be free from many objections—clean, cheap, and accurate.

Human milk contains more lactose and less caseinogen than cow's milk, the fat in the two cases only differing in a very slight amount. In converting cow's milk into humanised milk, the excess of caseinogen in the former is conveniently modified by dilution with whey, because whey contains all the constituents of milk except caseinogen, and the fat can previously be removed from the milk that is going to be converted into whey by the process of skimming. What remains then is to obtain the proper amounts of the various constituents, so as to have a result as nearly as possible identical with mothers' milk, and also to employ our methods in the best and simplest way possible.

I believe that home-made humanised milk is much superior to the dairy-made article. Cleanliness and constancy of composition are ensured: two very important factors in successful infant feeding. Expense is also lessened. Dairy-made humanised milk costs 8d. a pint, while the home-made article costs about 1d. a pint over the ordinary price of cow's milk. The process takes about 20 minutes daily.

As regards the use of a rennet preparation the glycerine extracts are very uncertain in action, and the glycerine with which they are made is possibly not pure. I have had made a purified standardised rennet in powder form, a given quantity of which will always curdle a certain quantity of milk. I have had this made up with the other constituents mentioned below into compressed tablet form to ensure accuracy of dosage, and for convenience.

Cow's milk contains 19 grs., human milk 25 grs. to the ounce of lactose. When milk is curdled by the addition of rennet 75 per cent. is whey. This whey contains all the lactose that was in the original quantity of milk. Therefore, if we take 10 oz. of cow's milk and curdle it, the resulting $7\frac{1}{2}$ oz. of whey contain 190 grs. of lactose, and if another 10 oz. of uncurdled milk is added to this whey the resulting mixture will contain 380 grs. of lactose. Now $17\frac{1}{2}$ oz. of human milk contains 420 grs. of lactose, so that in making humanised milk there is a considerable deficiency of lactose (40 grs. in $17\frac{1}{2}$ oz.) which we must supply. Infants, however, assimilate better a mixture not quite so concentrated, and I usually order this $17\frac{1}{2}$ oz. which results from an original pint, to be made up to a pint again by the addition of more cow's milk. Lactose should always be used, not dextrose; it is easier of digestion, and besides it is the natural constituent. The same dilution will result in an almost identical amount of caseinogen in the mixture, which, in the two cases, exists in the proportion of about 5 to 3. The tablets, besides the 40 grs. of lactose and the rennet, contain 1·5 grs. of sodii bicarb. each. This is added to neutralise any lactic acid formation in hot weather. Each also contains 0·5 gr. calcii phosph. I have added this last on account of its great value in assisting tissue formation. When it is considered at what a disadvantage the hand-fed infant starts when compared with the breast-fed infant, we ought to take every possible opportunity of lessening that disadvantage, and I am sure that the results are decidedly better with its employment.

The tablets are put up in tubes; full directions are enclosed with each tube. They are as follows:—Take two half pints of cow's milk; boil one half and skim the other. Add the skim to the boiled half pint. Heat the skimmed half to 100° Fahr., and to it add one tablet previously crushed, and keep milk at about the same temperature, with occasional stirring for 20 minutes. Separate the curds from the whey, and then boil the whey for two minutes, and then add this to the other half pint of milk. To this mixture add five tablespoonfuls of boiled cow's milk. Scald all vessels with boiling water immediately before and after use. Mr. Felthouse, of 30 Highbury Park, has manufactuaed the tablets for me.

